# Neoadjuvant chemoradiotherapy for patients with unresectable radically locally advanced colon cancer: a potential improvement to overall survival and decrease to multivisceral resection

**DOI:** 10.1186/s12885-021-07894-6

**Published:** 2021-02-19

**Authors:** Yan Yuan, Wei-Wei Xiao, Wei-Hao Xie, Pei-Qiang Cai, Qiao-Xuan Wang, Hui Chang, Bao-Qing Chen, Wen-Hao Zhou, Zhi-Fan Zeng, Xiao-Jun Wu, Qing Liu, Li-Ren Li, Rong Zhang, Yuan-Hong Gao

**Affiliations:** 1 State Key Laboratory of Oncology in South China, Collaborative Innovation Center for Cancer Medicine, Guangzhou, China; 2 Department of Radiation Oncology, Sun Yat-sen University Cancer Center, Guangzhou, China; 3 Departments of Medical Imaging and Interventional Radiology, Sun Yat-sen University Cancer Center, Guangzhou, PR China; 4 Department of Colorectal Surgery, Sun Yat-sen University Cancer Center, Guangzhou, PR China; 5 Department of Epidemiology and Biostatistics, Sun Yat-sen University Cancer Center, Guangzhou, PR China; 6 Department of Endoscopy and Laser, Sun Yat-sen University Cancer Center, Guangzhou, PR China

**Keywords:** Locally advanced colon cancer, Neoadjuvant chemoradiotherapy, Organ preservation, Pathological complete response

## Abstract

**Background:**

The management of unresectable locally advanced colon cancer (LACC) remains controversial, as resection is not feasible. The goal of this study was to evaluate the treatment outcomes and toxicity of neoadjuvant chemoradiotherapy (NACRT) followed with surgery and adjuvant chemotherapy in patients with unresectable radically LACC.

**Methods:**

We included patients who were diagnosed at our institution, 2010–2018. The neoadjuvant regimen consisted of radiotherapy and capecitabine/ 5-fluorouracil-based chemotherapy.

**Results:**

One hundred patients were identified. The median follow-up time was 32 months. The R0 resection rate, adjusted nonmultivisceral resection rate and bladder preservation rate were 83.0, 43.0 and 83.3%, respectively. The pCR and clinical-downstaging rates were 18, and 81.0%%, respectively. The 3-year PFS and OS rates for all patients were 68.6 and 82.1%, respectively. Seventeen patients developed grade 3–4 myelosuppression, which was the most common adverse event observed after NACRT. Tumor perforation occurred in 3 patients during NACRT. The incidence of grade 3–4 surgery-related complications was 7.0%. Postoperative anastomotic leakage was observed in 3 patients.

**Conclusions:**

NACRT followed by surgery was feasible and safe for selected patients with LACC, and can be used as a conversion treatment to achieve satisfactory downstaging, long-term survival and quality of life, with acceptable toxicities.

**Supplementary Information:**

The online version contains supplementary material available at 10.1186/s12885-021-07894-6.

## Introduction

Colon cancer is one of the most common cancers worldwide and accounted for approximately 6.1% of newly diagnosed cancers and 5.8% cancer-related deaths in 2018 [1]. Approximately 26% of patients with colon cancer present with a locally advanced disease [2]. In patients with locally advanced colon cancer, including patients with high-risk stage II or stage III disease, surgery and adjuvant chemotherapy are the standard treatments [3]. However, R0 resection is unable to be achieved in some patients with T4b, M0 or N2, M0 disease, even after multivisceral resection (MVR) [4]. Incomplete resection has not been shown to be beneficial [5]. Therefore, converting unresectable LACC to achieve radical resection may be essential to improve the quality of life and prolong the survival time of patients.

Neoadjuvant chemoradiotherapy (NACRT) has been well established as the standard therapy for locally advanced rectal cancer (LARC), and is related to a survival benefit and organ preservation [6]. The pathogenesis of colon and rectal cancer is similar. Thus, NACRT is worthy of investigation in patients with unresectable LACC. Actually, several reports with a small sample size have evaluated NACRT followed by radical surgery for LACC [7]. We also previously published several studies with small sample sizes [8, 9]. Results suggest that patients with LACC may benefit from NACRT. In the present study, we described the results of the administration of NACRT to patients with LACC over the last decade at a comprehensive cancer center.

## Methods

### Patient population and staging system

**Fig. 1:**
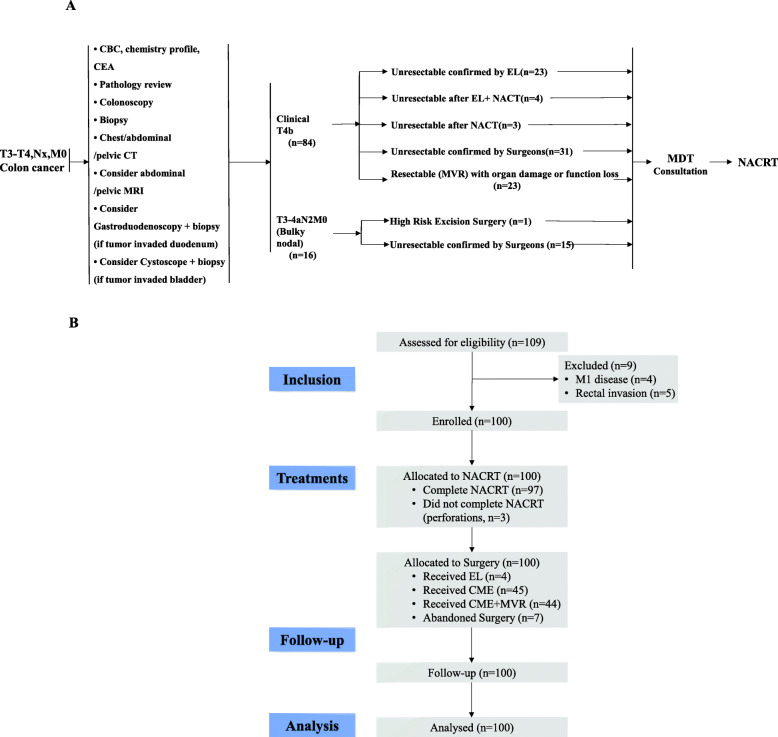
Flowchart of diagnosis of unresectable LACC (**a**) and Study profile (**b**). NACRT, Neoadjuvant chemoradiotherapy; EL, exploratory laparotomy; CME, complete mesocolic excision; MVR, multivisceral resection

The study was designed to evaluate the value of the NACRT for patients with unresectable LACC. This observational study was approved by our institutional medical ethics committee (B2020–063-01). One hundred eligible patients were identified who were diagnosed at our hospital from November 1, 2010 to June 31, 2018. Patients were selected to undergo NACRT on a case-by-case basis through a consultation with a multidisciplinary team (MDT). The pretreatment evaluation, the definition of unresectability and key exclusion criteria were described in our previous reports [8, 9]. Patients with LACC (defined as the primary tumor having an inferior margin ≥15 cm from the anal verge, as determined by colonoscopy) were candidates for NACRT if they met the criteria listed in the Fig. 1a. Patients’ medical records were reviewed and demographic, oncological, and pathological information was collected. A Charlson comorbidity index score was generated for each patient after a review of the medical history [10]. All patients have signed the informed consent form before treatment.

The pretreatment clinical stage of all patients treated before 2017 were re-evaluated according to the 8th edition of the Union for American Joint Cancer Committee (AJCC) TNM staging system. Then the 8th edition of TNM staging was used for clinical staging in patients who were diagnosed after 2017. Patients with peritoneal carcinomatosis classified as M1c were excluded from this study. Chest/abdomen/pelvic computed tomography (CT), pelvic magnetic resonance image (MRI), serum chemical profile including carcinoembryonic antigen (CEA), and considering colonoscopy were performed after NACRT to assess the response of tumors.

### Treatments and follow-up

Chemotherapy and radiotherapy, and surgery were described in detail in our previous reports [8]. Standard complete mesocolic excision (CME) would be performed after NACRT. When tumor infiltration or adhesion to the adjacent organs was detected intraoperatively, MVR was required. Radial margins were evaluated based on the pathological review principles of the NCCN guidelines. The formalin-fixed paraffin-embedded blocks of surgical samples from a portion of patients were tested for the MMR status using immunohistochemical staining for MLH1, MSH2, MSH6, and PMS2. Acute adverse events that occurred during or 30 days after NACRT were graded according to the Common Terminology Criteria for Adverse Events (CTCAE) (version 4.03). Postoperative complications were assessed according to the Clavien-Dindo classification [11].

Follow-up visits were performed by a multidisciplinary team every 3 months in the first 2 years after treatment, every 6 months in the following 3 years, and then yearly thereafter. Afterwards the patients were followed by an outpatient interview or household registration system. The last follow-up time point was December 31, 2019.

### Statistical analysis

Descriptive statistics were used to report patient, tumor, and treatment characteristics. Continuous data are presented as medians with interquartile ranges (IQRs). Categorical data are presented as numbers with percentages (%). Progression-free survival (PFS) was calculated from the initial diagnosis to the first disease progression or death. Overall survival (OS) was calculated from the diagnosis to the date of death. Disease-free survival (DFS) was calculated from the initial diagnosis to the first disease progression or death in the R0 group. The local recurrence (LR) rate was calculated in the R0 group. The distant metastasis (DM) rate was evaluated in all patients. The survival analysis was performed using R version 3.6.0. The packages ‘survival’ and ‘survminer’ were used for the survival analysis. Survival curves were also constructed by using R version 3.6.0. The multicollinearity regressions, the correlation matrix analysis, the Cox regression analysis, the Cox proportional hazard regression model were performed using STATA software (version 15). The multicollinearity regressions and a correlation matrix analysis were used to calculate the correlations between variables. Univariate and multivariate analyses were conducted to identify prognostic factors. A Cox regression analysis was used for univariate and multivariate analyses. The Cox proportional hazard regression model was used to estimate the hazard ratio (and corresponding 95% confidence interval [CI]) for each of the potential risk factors. A two-sided *P*-value < 0.05 was considered statistically significant.

## Results

### Characteristics and compliance

The study profile is shown in Fig. 1b. Patient characteristics, tumor staging and treatment details of the 100 patients with unresectable LACC are listed in Table 1. The pathological type of all patients was adenocarcinoma. All patients underwent colonoscopy and pathological biopsy at diagnosis. Eighty-four patients (84.0%) were diagnosed with stage cT4b tumors. The most common tumor location was the sigmoid colon (60/100, 60.0%), and bladder was the most commonly invaded organ (42/100, 42.0%). Fourteen (14/100, 14.0%) of these patients had a bladder fistula caused by tumor infiltration prior to the treatments. Meanwhile, 19 (19/100, 19.0%) patients had an intestinal obstruction at diagnosis. All patients received NACRT after the remission of the obstruction. Prophylactic enterostomy was performed in 27 (27/100, 27.0%) patients.

**Table 1:** Baseline pathological and clinical characteristics of patients

	No. (%)
**Age, median, years**	**54 (43–63)**
**≤65**	**84 (84.0%)**
**> 65**	**16 (16.0%)**
**Sex**	
**Male**	**73 (73.0%)**
**Female**	**27 (27.0%)**
**KPS**	
**90**	**78 (78.0%)**
**80**	**22 (22.0%)**
**BMI, median,**	**21.4 (19.5–23.6)**
**Primary tumor length, median, cm**	**7.4 (1.7–8.9)**
**Primary tumor location**	
**Sigmoid colon**	**60 (60.0%)**
**Descending colon**	**3 (3.0%)**
**Transverse colon**	**10 (10.0%)**
**Ascending colon**	**25 (25.0%)**
**Ileocecus**	**2 (2.0%)**
**Tumor differentiation**	
**High**	**20 (20.0%)**
**Moderate**	**65 (65.0%)**
**Low**	**15 (15.0%)**
**CEA pre-CRT, median, ng/ml**	**6.1 (3.0–21.3)**
**Complication**	
**No**	**56 (56.0%)**
**Yes**	**44 (44.0%)**
**cT stage**	
**T3**	**4 (4.0%)**
**T4a**	**12 (12.0%)**
**T4b**	**84 (84.0%)**
**cN stage**	
**N0**	**1 (1.0%)**
**N1**	**36 (36.0%)**
**N2**	**63 (63.0%)**
**Clinical stage**	
**IIC**	**1 (1.0%)**
**IIIB**	**16 (16.0%)**
**IIIC**	**83 (83.0%)**
**Involved organ**	
**Bladder**	**42 (42.0%)**
**Ureter**	**12 (12.0%)**
**Renal and perirenal fat, prerenal space**	**6 (6.0%)**
**Pelvic wall**	**10 (10.0%)**
**Presacral space**	**2 (2.0%)**
**Abdominal wall**	**11 (11.0%)**
**Mesentery**	**2 (2.0%)**
**Great vessel**	**8 (8.0%)**
**Small intestine**	**26 (26.0%)**
**Greater omentum**	**5 (5.0%)**
**Gallbladder**	**6 (6.0%)**
**Liver**	**9 (9.0%)**
**Appendix**	**1 (1.0%)**
**Pancreas**	**2 (2.0%)**
**Uterus**	**6 (6.0%)**
**Vagina**	**1 (1.0%)**
**Seminal vesicle gland**	**3 (3.0%)**
**Vas deferens**	**2 (2.0%)**
**Iliopsoas muscle**	**1 (1.0%)**
**Bladder fistula/perforation**	
**Yes**	**14 (14.0%)**
**No**	**86 (86.0%)**
**Intestinal obstruction**	
**Yes**	**19 (19.0%)**
**No**	**81 (81.0%)**
**Family history**	
**Yes**	**20 (20.0%)**
**No**	**80 (80.0%)**
**Charlson Comorbidity Score**	
**0**	**82 (82.0%)**
**1**	**15 (15.0%)**
**2**	**2 (2.0%)**
**3**	**1 (1.0%)**
**MMR**	
**dMMR**	**14 (22.6%)**
**pMMR**	**48 (77.4%)**
**Unknown**	**38**

*Abbreviations*: *KPS* Karnofsky Performance Status, *BMI* Body Mass Index, *cT stage* Clinical T stage, *cN stage* Clinical N stage, *MMR* Mismatch repair phenotype

Neoadjuvant chemotherapy was a capecitabine/ 5FU-based regimen with a median cycles number of 4 (IQR 3–4). The sketching methods of GTV and CTV are described in our previous two articles [8, 9]. The dose of GTV is 46-54Gy/23–27 fractions. The dose of CTV is 41.4–46 Gy/23–27 fractions. Three patients developed colon cancer perforation during NACRT. Ninety-seven patients successfully received the allocated NACRT. 93 (93/100, 93.0%) patients underwent surgical treatment, and the other 7 (7/100, 7.0%) patients abandoned further surgical treatment after NACRT with treatment details shown in Supplementary Table 1.

### Short-term efficacy

Among all patients receiving NACRT, 93 (93/100, 93.0%) patients underwent surgery with the goal of a radical operation (Supplementary Figure 1). The details of operations and pathological findings from the 93 patients are presented in the Table 2. In this cohort study, 89.25% (83/93) of the 93 patients who underwent surgery reached R0, and 81.0% (81/100) patients achieved downstaging after NACRT. The pCR rate was 18.0% (18/100).

**Table 2:** Treatment outcomes of operations and pathological findings

	No. (%)
**CEA preoperative, median, ng/ml**	**2.4 (1.5–4.1)**
**Surgery situation**	
**R0**	**83 (89.2%)**
**R2**	**6 (6.5%)**^**b**^
**EL**^**a**^	**4 (4.3%)**
**Downstage T**	
**Yes**	**65 (69.9%)**
**No**	**28 (30.1%)**^**c**^
**Downstage N**	
**Yes**	**84 (90.3%)**
**No**	**9 (9.7%)**^**c**^
**Downstage**	
**Yes**	**81 (87.1%)**
**No**	**12 (12.9%)**^**c**^
**MVR**	
**Yes**	**44 (47.3%)**
**No**	**49 (52.7%)**^**c**^
**pCR**	
**Yes**	**18 (19.4%)**
**No**	**75 (80.6%)**^**c**^
**Resection Surgery-Radiotherapy interval Median, d (range)**	**63.0 (55–76.5)**

*Abbreviations*: *ypT stage* Postoperative pathological T stage, *ypN stage* Postoperative pathological N stage, *MVR* Multivisceral resection, *pCR* Pathological complete remission

^a^EL; ^b^two patients who had perforations during NACRT were included in this group; ^c^four patients who underwent EL were included in this group

**Fig. 2:**
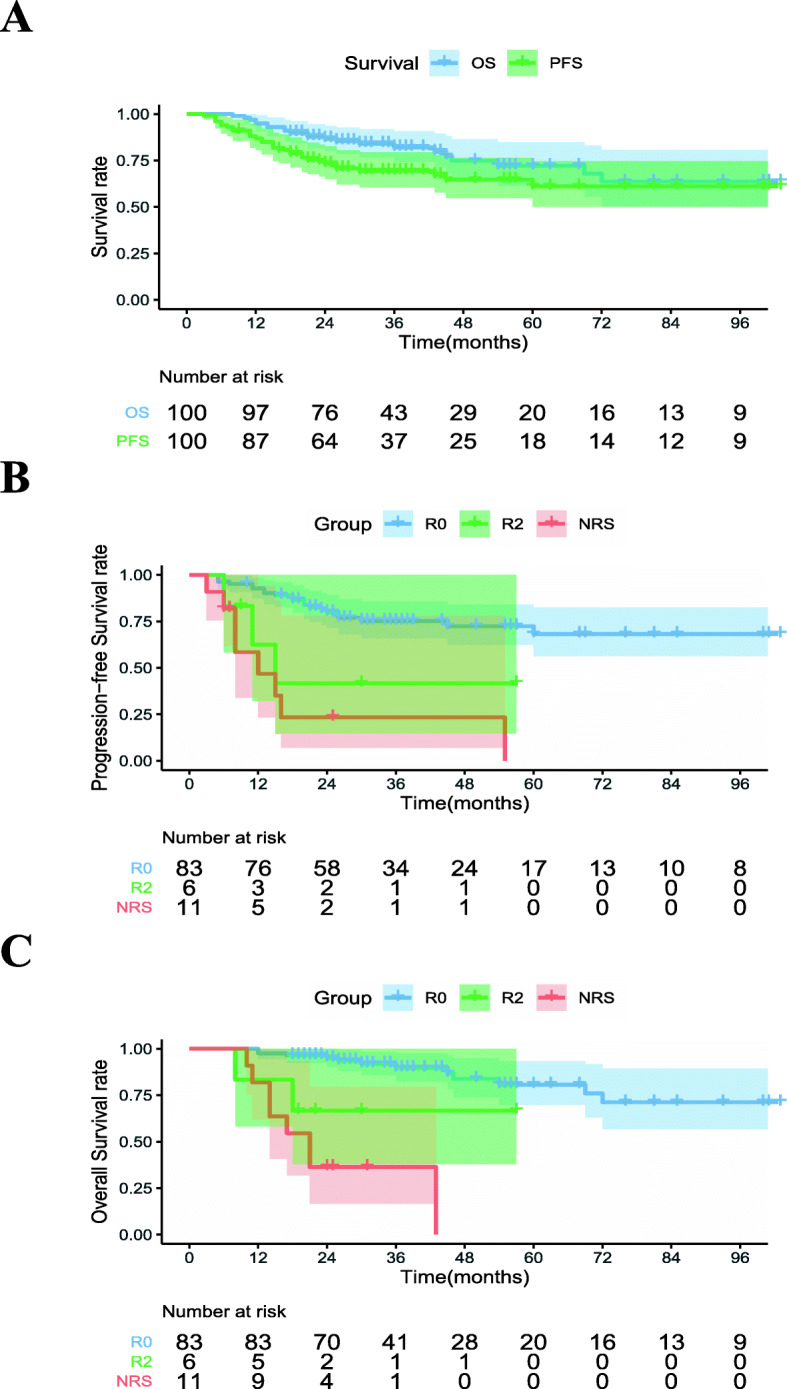
Changes in the imaging features of patients with T4b tumors after neoadjuvant radiotherapy and chemotherapy. **a** Imaging figures presented ascending colon cancer with invasion of ileum (yellow arrow) and lymph node metastasis with invasion of parietal peritoneum (blue arrow). After NACRT, ascending colon cancer and lymph node metastasis were obviously smaller than before NACRT; **b** Imaging figures showed sigmoid colon cancer with invasion of bladder (brown arrow), small intestinal (yellow arrow) and peritoneum (blue arrow). Sigmoid colon cancer shrank significantly after NACRT

Seventy (83.3%, 70/84) patients with cT4b diseases achieved R0 resection. 34.5% (29/84) patients underwent CME without MVR and 48.8% (41/84) patients required CME with MVR. The changes in the imaging features of cT4b patients after NACRT are shown in Fig. 2.

Among the 42 patients with pretreatment bladder invasion (Supplementary Figure 2A), 2 (2/42, 4.8%) and 22 (22/42, 52.4%) patients underwent total cystectomy and partial cystectomy respectively. 13 (13/42, 31.0%) patients did not have bladder resection. Five (5/42, 11.9%) patients did not undergo cystectomy due to unresectability or personal reasons. Therefore, totally 83.3% (35/42) patients retained bladder function.

In addition, the small intestine is the second most common site of adjacent organ invasion in patients with LACC (Supplementary Figure 2B). Nine (9/26, 34.6%) patients with R0 resection avoided small intestine resection. 7 (7/26, 26.9%) patients underwent small bowel resection and 4 (4/26, 15.4%) patients had a Whipple’s procedure. Six (6/26, 23.1%) patients did not received radical surgery because the conversion therapy failed or for personal reasons.

### Long-term survival

The median follow-up period of surviving patients was 32 (IQR 24–55) months in the entire group. Using the public security household registration system, we inquired about the survival outcomes of 6 patients who were lost medical follow-up. None of the patients were lost to follow-up.

**Fig. 3:**
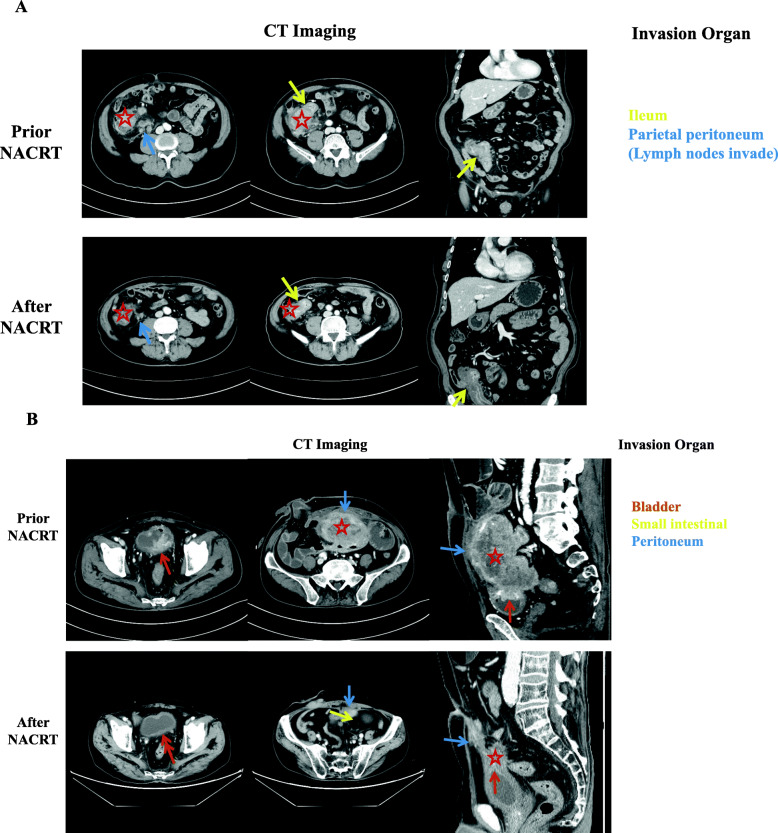
Survival curves. OS and PFS curves of all patients (**a**). PFS curves (**b**) and OS curves (**c**) curves of the patients with unresectable LACC by resection group (R0 vs R2 vs NRS). P values were calculated from the comparison between the groups. NRS: Nonresectable surgery

The estimated PFS rate at 3 years was 68.6% for the whole group (Fig. 3a). The estimated OS rate at 3 years was 82.1% (Fig. 3a). In this study, local control failed in 16 (16/100, 16.0%) patient, and DM occurred in 28 (28/100, 28.0%) patients. Among the patients who underwent R0 surgery, the 3-year PFS, OS, LR and DM rates were 74.0% (Fig. 3b), 89.6% (Fig. 3c), 13.4 and 20.8%, respectively. As expect, the LR rate and DM rate of 17 patients with non-R0 resection were disappointing. The cumulative 3 year PFS, OS, local progression, DM rates for this cohort were 38.1, 45.8, 71.9, and 41.2%, respectively.

In the univariate analysis (Supplementary Table 3), low differentiation, non-R0 resection, ypT stage (ypT4a-T4b) and advanced ypTNM stage (ypIIb-IIIc) were significantly associated with poor OS and PFS in the whole group (Supplementary Figure 3A-H), and a low KPS, low differentiation, and VLPNI (vascular or lymphatic or perineural invasion) positively were associated with a poor DFS in the R0 group (Supplementary Figure 3I-K). Both the results of multicollinearity regression (Supplementary Table 4.1) and the correlation matrix (Supplementary Table 4.2) identified strong correlations between two variables (ypT stage group and ypTNM stage group). Then we subjected the ypTNM stage group to the multivariate analysis. In the multivariate analysis, differentiation remained an independent prognostic factor for OS rates (Table 3). However, no difference in survival was observed between patients with different ypN stages, MMR status, genders, ages and nutritional statuses. A Low TRG score seems to be associated with the poor OS but failed to reach the significance (Supplementary Figure 3M). We supposed that the number of each group was small to reach statistical difference.

**Table 3:** Multivariate Cox analysis of prognostic factors for OS, PFS, DFS. (OS: *p* = 0·0006, χ2 = 27·27; PFS: *p* = 0·065, χ2 = 19·35; DFS: *p* = 0·087, χ2 = 9·62)

	OS		PFS		DFS	
	HR (95%CI)	***p***	HR (95%CI)	***p***	HR (95%CI)	***p***
**KPS (80**^**a**^ **vs 90)**	**··**	**··**	**0.50 (0.20–1.22)**	**0.13**	**0.53 (0.21–1.36)**	**0.2**
**Complication (yes**^**a**^ **vs no)**	**1.70 (0.54–5.41)**	**0.37**	**1·56 (0·67–3·64)**	**0.3**	**··**	**··**
**Differentiation**	**··**	**0**	**··**	**0.06**	**··**	**0.6**
**High**^**a**^	**1**	**··**	**1**	**··**	**1**	**··**
**Middle**	**3.07 (0.29–32.59)**	**0.35**	**1.60 (0.31–8.18)**	**0.57**	**1.48 (0.32–6.82)**	**0.6**
**Low**	**26.87 (2.38–303.60)**	**0.01**	**4.75 (0.73–22.74)**	**0.07**	**2.39 (0.42–13.51)**	**0.3**
**ypN stage (N0**^**a**^ **vs N1 + N2)**	**0.31 (0.029–3.19)**	**0.32**	**0.41 (0.073–2.24)**	**0.3**	**··**	**··**
**ypTNM stage (0-IIB**^**a**^ **vs IIC-IIIC)**	**3.44 (0.82–14.38)**	**0.09**	**1.82 (0.67–4.99)**	**0.24**	**1.49 (0.53–4.16)**	**0.5**
**pCR (yes**^**a**^ **vs non)**	**0.40 (0.053–2.99)**	**0.37**	**1.18 (0.29–4.84)**	**0.81**	**··**	**··**
**MVR (non**^**a**^ **vs yes)**	**1.07 (0.28–4.14)**	**0.92**	**0.97 (0.40–2.36)**	**0.95**	**··**	**··**
**VLPNI (negative**^**a**^ **vs positive)**	**3.61 (0.84–15.54)**	**0.09**	**2.16 (0.77–6.02)**	**0.14**	**2.01 (0.64–6.37)**	**0.2**
**R0 resection (non-R0**^**a**^ **vs R0)**	**0.21 (0.030–1.48)**	**0.12**	**0.60 (0.12–3.08)**	**0.54**	**··**	**··**

*Abbreviations*: *KPS* Karnofsky Performance Status, *cT stage* Clinical T stage, *MVR* Multivisceral resection, *pCR* Pathologic complete remission, *VLPNI* Vascular or lymphatic or perineural invasion

^a^The control group of multivariate Cox analysis

The information about the treatment-related toxicities is shown in Table 4. Based on the CTCAE criteria ver. 4.03, the most common grade 3 to 4 NACRT-related toxicities were myelosuppression, gastrointestinal (GI) toxicities and mucositis/dermatitis. The incidence rates were 17.0, 7.0, and 3.0%, respectively. Eight patients developed an intestinal obstruction during NACRT. Seven patients developed diarrhea and abdominal pain in the process of radiotherapy. Only 3 patients did not complete the radiation course due to tumor rupture, of which two patients underwent emergency surgery and one patient requested for supportive treatment instead of the operation as described before. No patients died during NACRT.

**Table 4:** Toxicities of NACRT and complications of surgery

	No. (%)
**Myelosuppression**	
**Grade 0–2**	**83 (83.0%)**
**Grade 3–4**	**17 (17.0%)**
**Mucositis/dermatitis**	
**Grade 0–2**	**97 (97.0%)**
**Grade 3–4**	**3 (3.0%)**
**GI toxicities**	
**Grade 0–2**	**93 (93.0%)**
**Grade 3–4**	**7 (7.0%)**
**Intestinal obstruction**	
**Yes**	**8 (8.0%)**
**No**	**92 (92.0%)**
**Postsurgical complications**	
**Grade 0–2**	**82 (92.1%)**
**Grade 3–4**	**7 (7.9%)**
**Nonresectable surgery**^**a**^	**11**
**Anastomotic leakage**	
**Yes**	**3 (3.2%)**
**No**	**86 (96.8%)**
**Nonresectable surgery**^**a**^	**11**

*Abbreviation*: *GI* Gastrointestinal

^a^Includes 7 patients who abandoned surgery and 4 patients who underwent EL

Among the 93 patients who underwent surgery, grade 3/4 Clavien-Dindo postsurgical complications were observed in 7 patients (7/100, 7.0%). In addition, 3 patients experienced anastomotic leakage after radical surgery, but completely recovered after the enterostomy or the repair of fistula.

## Discussion

Worldwide, surgery is the cornerstone of curative treatment for the colorectal cancer. Radical resection is one of the most important predictors of LR and long-term survival in patients with stage III colon cancer [12]. Hence, a crucial question is whether patients with LACC are able to be converted from an unresectable status to resectable status with the goal of a cure. For locally unresectable radically or bulky nodal disease or clinical T4b colon cancer, neoadjuvant chemotherapy is recommended by NCCN guidelines [13]. In the present study, 7 patients with unresectable LACC underwent 3–5 courses of chemotherapy had stable diseases before NACRT and failed to convert to resection surgery. Therefore, more effective treatments are urgently needed.

Previously published studies have showed that NACRT is an effective choice for patient with unresectable LACC. The evidence of the effectiveness of NACRT for colon cancer is still accumulating. Between 2000 and 2010, several single or very small sample size case reports described the use of NACRT for colon cancer [14, 15]. Taylor et al. retrospectively analyzed 25 patients with LACC who were treated with en-bloc surgical resection with radiotherapy and chemotherapy. Those patients had a median survival of 38.2 months and a 5-year survival rate of 49% [16]. Since 2010, a greater number of reports have investigated small sample sizes. The application of NACRT followed by MVR to 33 patients with primary locally advanced adherent colon cancer and 15 patients with locally recurrent adherent colon cancer patients both achieved high rates of R0 resection and excellent LR in studies published in 2012 and 2014, respectively, by Wong et al’ team from Canada [17, 18] (Supplementary Table 2). In Taiwan, Chun-Ming Huang et al. delivered NACRT to 36 patients with potentially incomplete resection of LACC, as defined by the presence of a T3 tumor with extramural extension of > 5 mm or a T4 tumor diagnosed by imaging. Approximately 26.4% of patients achieved pCR and the 2-year estimated OS and DFS rates were 88.7 and 73.6%, respectively [19].

Because the benefits of NACRT for colon cancer are not clear, the MDT of our cancer center began to explore the application of NACRT only in patients with LACC. We have previously also reported the treatment outcomes of 21 and 60 patients with unresectable radically LACC in 2016 and 2018, respectively. The current report describes 100 patients enrolled from 2010 to 2018 and is the largest sample in which NACRT for LACC has been analyzed. Similar to previous studies, NACRT results in downstaging of tumor for patients with LACC, in which the pCR rate is 18%. The low rate of pCR in the Canadian studies [17] may be due to the use of 5FU alone in NACRT, while we used double chemotherapy during NACRT, similar to the study by Huang CM et al. [19]. Doublet chemotherapy was well tolerated in the patients receiving NACRT for LACC and may contribute to better tumor regression, as observed in patients with LARC [20]. The higher R0 resection rate of LACC in our study may translate to long-term survival benefit. The 3-year PFS and OS rates were 68.6 and 82.1%, respectively, similar to two previous studies [8, 9], and significantly higher than rates reported in the literatures for patients undergoing neoadjuvant chemotherapy [21]. Recently, a research team has compared the treatment outcomes of cT4 colon cancers treated with neoadjuvant radiotherapy (NRT) to patients treated without NRT in their tertiary care center and the National Cancer Database [22, 23]. Both studies showed that the 5-year OS rate of the NRT group was 20 to 25% higher than that of the non-NRT group, and even when patients with cT4b tumors received NRT than non-NRT. Thus, the survival benefit of NACRT is plausible for patients with cT4 colon cancer.

Univariate and multivariate survival analyses revealed independent association of pathological grade with OS. The results were consistent with findings from the study by Wang el at, who constructed a prediction model to predict cause-specific death in elderly patients with colorectal cancer after surgery, particularly for patients with colon cancer [24]. By performing subgroup analysis, patients with better T downstaging had higher survival rates in the present study (Supplementary Figure 3E). Similar results were obtained from the analysis of ypTNM staging (Supplementary Figure 3G). Meanwhile, survival was prolonged in the patients who achieved pCR than in the patients who did not achieve pCR after NACRT. However, the difference was not statistically significant and may be caused by the small sample sizes.

The incidence of acute toxicities of NACRT is another important concern. Bone marrow toxicity was the most common adverse event, of which the incidence grade 3–4 adverse events was 17.0%. The incidences of severe gastrointestinal and skin reactions were similar to patients who received neoadjuvant chemotherapy for LACC [25]. According to previous studies, IMRT accurately delivers radiation to tumors and decreases the dose administered to normal tissues [26]. In our study, 97 (97.0%) patients successfully completed the full doses of radiotherapy. Three patients (3.0%) experienced tumor perforation during the course of NACRT. We speculated that the tumor perforation may be attributed to the radiotherapy, which may cause rapid tumor regression. Therefore, based on the results of the present study, NACRT for colon cancer is safe and tolerable.

In Canadian studies, all patients underwent MVR with a relatively high incidence (> 30%) of postoperative complications [17, 18, 27]. The incidence of postsurgical complications in our series was lower, namely, 7.0%. And no significant difference in survival existed between the MVR group and non-MVR group (Supplementary Fig 3L). Hence, we consider that NACRT can reduce the probability of MVR and decrease the incidence of postoperative complications.

As is well known that the preservation of bladder function is of vital importance to patients in terms of quality of life [28]. In our study, the bladder function was retained in 30.95% of patients with primary bladder invasion. Similarly, several adjacent organs avoided surgical resection or the scope of surgical resection was reduced, such as the small intestine, duodenum, kidney, liver, pancreas and large blood vessels. Therefore, this treatment strategy is more conducive to the preservation of organs and functions, with the results of an improvement in the survival rate and an improvement in the quality of life.

Sensitivity of the deficient mismatch repair (dMMR) phenotype to conventional chemotherapy and radiotherapy is still controversial [29]. At the ASCO conference in 2019, Matt et al. presented an oral report stating that the rate of tumor regression after neoadjuvant chemotherapy markedly was reduced in patient with dMMR tumors, while the rate of pCR was similar. In our study, patients with the dMMR phenotype had similar survival rates to patients with the pMMR phenotype (*p* = 0.880). Notably, dMMR tumors present opportunities for immunotherapy [30]. Further studies are needed to determine whether NACRT combined with immunotherapy improves the prognosis of patients with dMMR tumors, and we initiated a phase II clinical trial (NCT04301557).

The limitations of the study are the nature of nonrandomized study, moderate sample size and the follow-up time was not long enough. In addition, the specific strategies of NACRT for patients with unresectable LACC enrolled in our study were inconsistent. The optimal strategy of NACRT for LACC requires further investigation. Therefore, we are conducting an open multicenter, randomized controlled trial to further validate the results by comparing the efficacy of neoadjuvant chemotherapy and NACRT in patients with unresectable LACC (NCT03970694). To date, 25 patients have been recruited and the prospective data are expected, which may provide the higher levels of evidence.

In conclusion, NACRT might provide an opportunity for patients with unresectable LACC to achieve R0 resection, which might translate into a survival benefit and better quality of life.

## Supplementary Information

**Additional file 1: Supplementary Figure 1.** The flowchart of treatment of unresectable LACC. Abbreviations: MVR, multivisceral resection; EL, exploratory laparotomy; CME, complete mesocolic excision. **Supplementary Figure 2.** The surgical details of adjacent organs. Bladder (A); Small intestine (B). **Supplementary Figure 3.** Subgroup analysis of survival. OS analyzed in patients with unresectable LACC treated with NACRT and surgery by differentiation (A), Resection group (C), ypT stage (E), ypTNM stage (G), MVR (L), TRG score (M). PFS analyzed in all patients by differentiation (B), resection group (D), ypT stage (F), ypTNM stage (H). DFS analyzed in patients with radical surgery by KPS (I), Differentiation (J), VPLNI (K). *P* values in the figure were calculated from the comparison of the groups. NRS: Nonresectable surgery. **Supplementary Table 1.** Tumor characteristics and treatment of patients who abandoned surgery. **Supplementary Table 2.** Characteristics of studies included in the discussion. **Supplementary Table 3.** Univariate Cox analysis of prognostic factors for OS, PFS, DFS. **Supplementary Table 4.** 1. Multiple linear regression coefficients. 2. Correlation matrix analysis.

## Data Availability

All data are available via the corresponding author.

## Abbreviations

LACC: Locally advanced colon cancer
NACRT: Neoadjuvant chemoradiotherapy
MVR: Multivisceral resection
MDT: Multidisciplinary team
AJCC: American Joint Cancer Committee
CT: Computed tomography
MRI: Magnetic resonance image
CEA: Carcinoembryonic antigen
CME: Complete mesocolic excision
CTCAE: Common Terminology Criteria for Adverse Events
IQRs: Interquartile ranges
PFS: Progression-free survival
OS: Overall survival
DFS: Disease-free survival
LR: Local recurrence
DM: Distant metastasis
CI: Confidence interval
GI: Gastrointestinal
NRT: Neoadjuvant radiotherapy
dMMR: Deficient mismatch repair
