# Supplementary material for: Neoadjuvant chemoradiotherapy for patients with unresectable radically locally advanced colon cancer: a potential improvement to overall survival and decrease to multivisceral resection
Source: BMC Cancer. 2021 Feb 19;21:179. doi: 10.1186/s12885-021-07894-6 (PMC7893883; doi:10.1186/s12885-021-07894-6)
Supplement: Supplementary file 1 — Additional file 1: Supplementary Figure 1. The flowchart of treatment of unresectable LACC. Abbreviations: MVR, multivisceral resection; EL, exploratory laparotomy; CME, complete mesocolic excision. Supplementary Figure 2. The surgical details of adjacent organs. Bladder (A); Small intestine (B). Supplementary Figure 3. Subgroup analysis of survival. OS analyzed in patients with unresectable LACC treated with NACRT and surgery by differentiation (A), Resection group (C), ypT stage (E), ypTNM stage (G), MVR (L), TRG score (M). PFS analyzed in all patients by differentiation (B), resection group (D), ypT stage (F), ypTNM stage (H). DFS analyzed in patients with radical surgery by KPS (I), Differentiation (J), VPLNI (K). P values in the figure were calculated from the comparison of the groups. NRS: Nonresectable surgery. Supplementary Table 1. Tumor characteristics and treatment of patients who abandoned surgery. Supplementary Table 2. Characteristics of studies included in the discussion. Supplementary Table 3. Univariate Cox analysis of prognostic factors for OS, PFS, DFS. Supplementary Table 4. 1. Multiple linear regression coefficients. 2. Correlation matrix analysis. [file 12885_2021_7894_MOESM1_ESM.docx]

**Neoadjuvant chemoradiotherapy for patients with unresectable radically locally advanced colon cancer: a potential improvement to overall survival and decrease to multivisceral resection**

Yan Yuan^#1,2^, Wei-Wei Xiao^#1,2^, Wei-Hao Xie^#1,2^, Pei-Qiang Cai^1,3^, Qiao-Xuan Wang^1,2^, Hui Chang^1,2^, Bao-Qing Chen^1,2^, Wen-Hao Zhou^1, 4^, Zhi-Fan Zeng^1,2^, Xiao-Jun Wu^1, 4^, Qing Liu^1,5^, Li-Ren Li^1, 4^, Rong Zhang^*1,6^, Yuan-Hong Gao^*1,2^

^#^ These authors contributed equally to this work

^*^ These authors contributed equally to this work

Correspondence: Yuan-Hong Gao, Email: gaoyh@sysucc.org.cn

^1^State Key laboratory of Oncology in South China, Collaborative innovation Center for cancer Medicine, Guangzhou, China; ^2^ Department of Radiation Oncology, Sun Yat-sen University Cancer Center, Guangzhou, China; ^3^ Departments of Medical Imaging and Interventional Radiology, Sun Yat-sen University cancer center, Guangzhou, PR china; ^4^ Department of colorectal surgery, Sun Yat-sen University cancer center, Guangzhou, PR china; ^5^ Department of Epidemiology and Biostatistics, Sun Yat-sen University cancer center, Guangzhou, PR china; ^6^ Department of Endoscopy and Laser, Sun Yat-sen University cancer center, Guangzhou, PR china

**Supplementary materials**

**Supplementary Figure1. The flowchart of treatment of unresectable LACC.** Abbreviations: MVR, multivisceral resection; EL, exploratory laparotomy; CME, complete mesocolic excision.

**
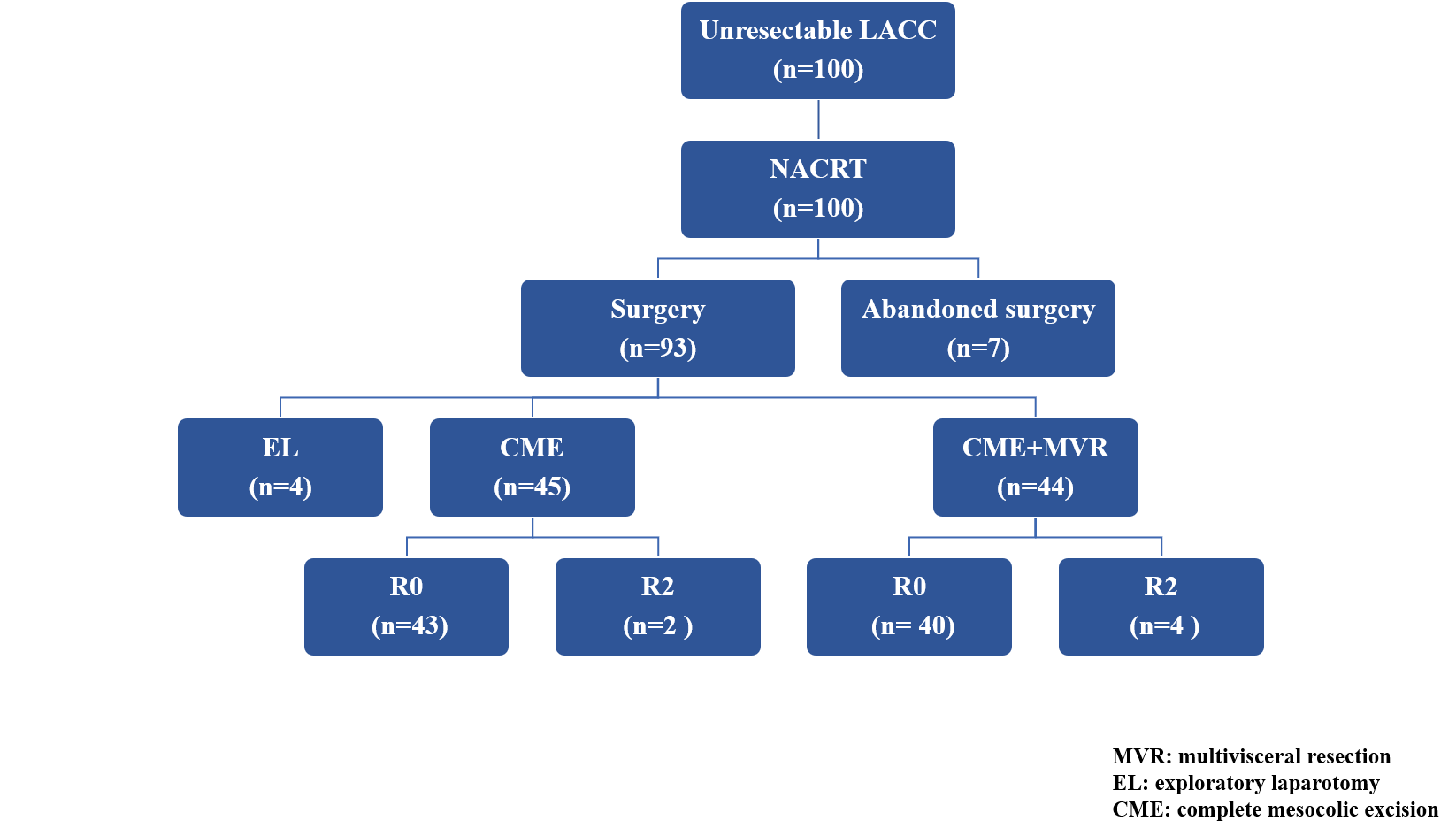
**

**Supplementary Figure 2. The surgical details of adjacent organs. Bladder(A); Small intestine(B)**

**
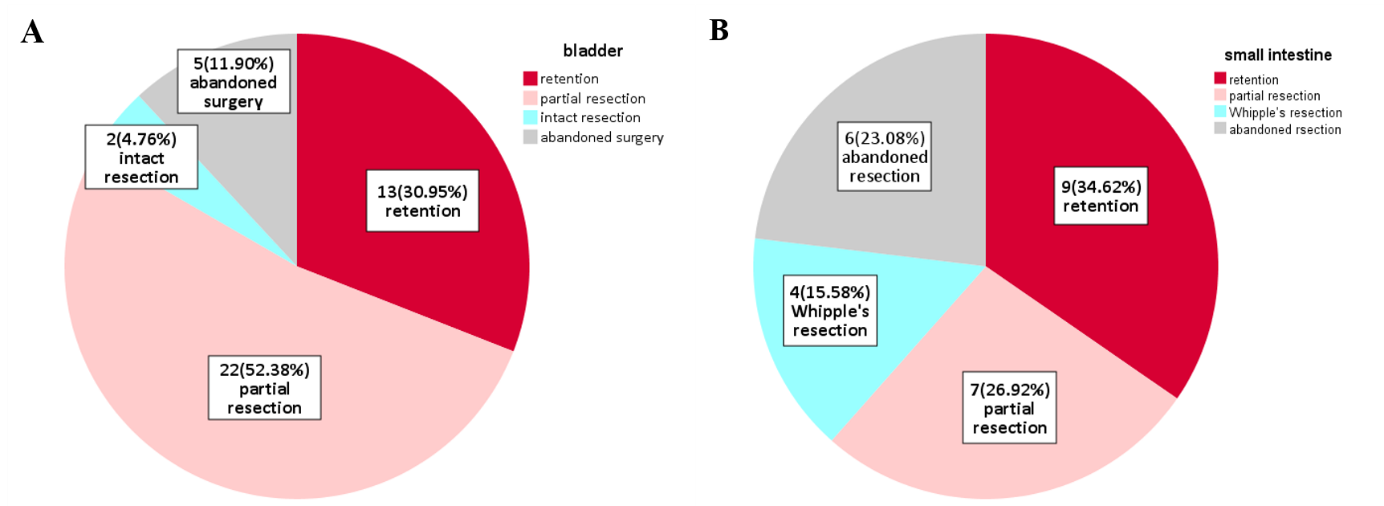
**

**Supplementary Figure 3. Subgroup analysis of survival.** OS analyzed in patients with unresectable LACC treated with NACRT and surgery by differentiation (A), Resection group (C), ypT stage (E), ypTNM stage (G), MVR (L), TRG score (M). PFS analyzed in all patients by differentiation(B), resection group (D), ypT stage (F), ypTNM stage (H). DFS analyzed in patients with radical surgery by KPS (I), Differentiation (J), VPLNI (K). P values in the figure were calculated from the comparison of the groups. NRS: Nonresectable surgery.


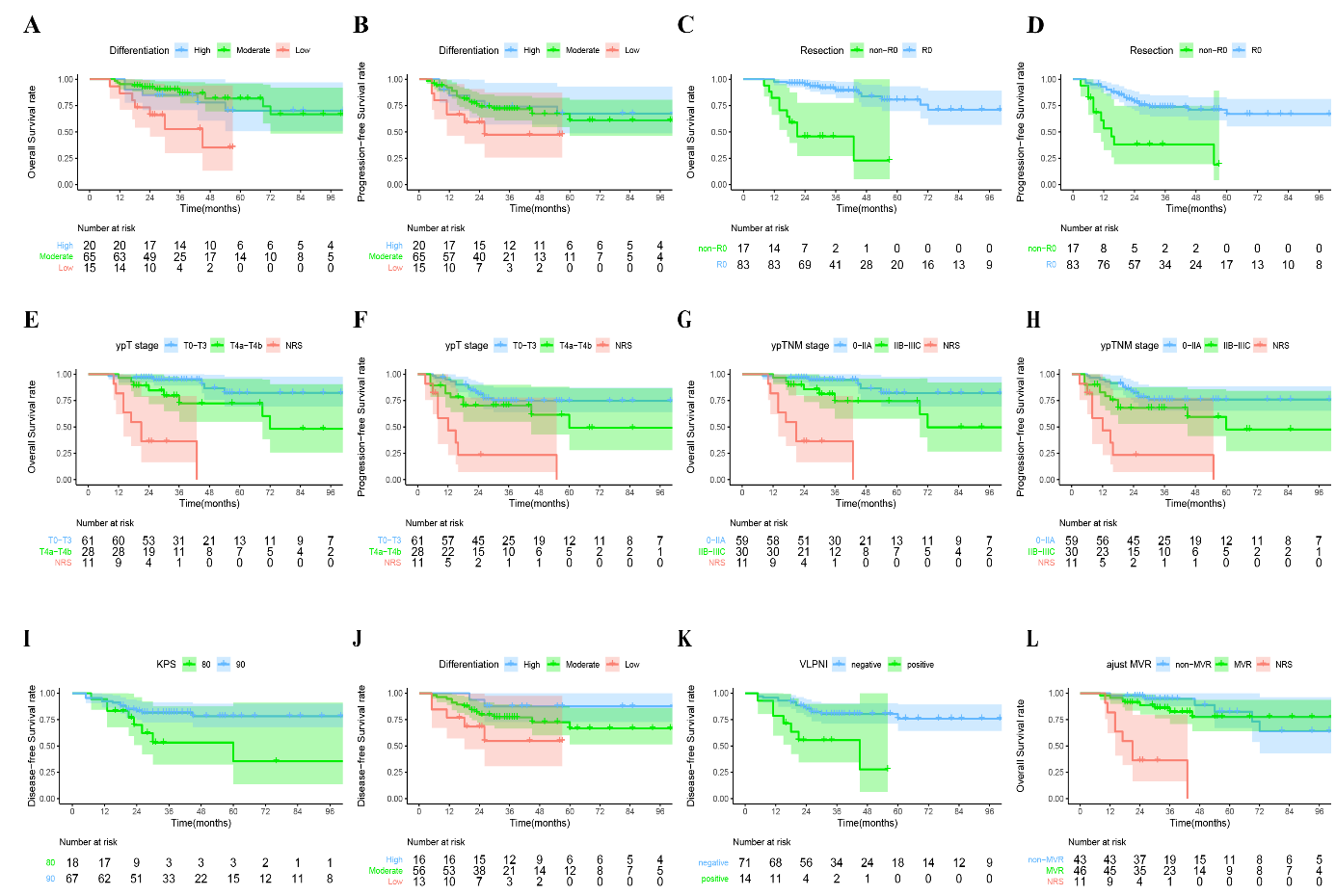


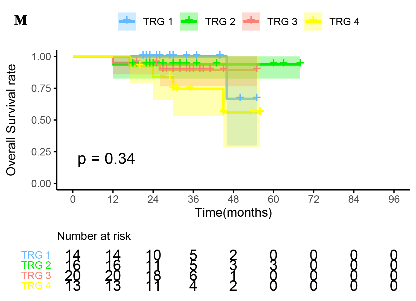


**Supplementary Tables.xls:**

**Supplementary Table 1.** Tumor characteristics and treatment of patients who abandoned surgery.

Abbreviations: F, female; M, male; RT, radiotherapy; OS, overall survival; ms, months; AWD, alive with disease; FOD, free of disease; DOD, dead of disease; cTNM, clinical tumor, node and metastasis stage; FOLFIRI, 5-fluorouracil, leucovorin, irinotecan; CAPEOX, capecitabine and oxaliplatin; 3D-CRT, three-dimensional conformal radiotherapy; VMAT, volumetric modulated arc therapy.

Notes: α The chemotherapy is presented as the regimen × the number of cycles.

& This patient was unable to surgery because of 4 grade myelosuppression after NACRT. After the blood analysis returned to normal after symptomatic treatment, the patient continued oral capecitabine chemotherapy for a total of 8 cycles. The repeat examination assessed the tumor as near-CCR after 16 months.

Reasons why patients refused surgery:

A The symptoms of the patients were significantly relieved after NACRT, and surgical treatment was feasible. However, patients refused radical surgery which may need extensive multiple resection surgeries, considering the risk of postoperative complications and sequelae.

B Personal privacy of patients.

C Grade 4 myelosuppression after NACRT.

| 7 | 6 | 5 | 4 | 3 | 2 | 1 | **case** |
| --- | --- | --- | --- | --- | --- | --- | --- |
| M/44 | F/68 | M/33 | F/64 | M/74 | M/41 | M/61 | **Sex/**  **age (years)** |
| cT4bN2M0 | cT4bN1M0 | cT4bN2M0 | cT4bN2M0 | cT4aN2M0 | cT4bN1M0 | cT4bN2M0 | **cTNM** |
| Ascending colon | Ascending colon | Ascending colon | Descending colon | Sigmoid | Sigmoid | Sigmoid | **Site** |
| duodenal/stomach/mesenteric vessels | liver | liver/kidney | kidney/  pancreas | No | abdominal wall/bladder/  spermatic cord | bladder/ureter | **Involved**  **structures** |
| gastrojejunostomy+ileotransversostomy | No | Intestinal colostomy | No | No | EL,  6xFOLFIRI | No | **Therapy prior to enrollment** |
| CAPEOX×4 | CAPEOX×2  +Capecitabine×4 | CAPEOX×2 | CAPEOX×2 | Capecitabine×2 | CAPEOX×3 | CAPEOX×7  +Capecitabine×1 | **Induction /Concurrent chemotherapy^α^** |
| VMAT,  50Gy/25F | VMAT,  47.5Gy/25F | VMAT,  20Gy/10F | VMAT,  20Gy/10F | 3D-CRT,  45Gy/23F | VMAT, 50Gy/25F | VMAT, 50Gy/25F | **RT (technique, dose)** |
| No | No | Yes | Yes | yes | yes | yes | **Suitable for surgery after NRT** |
| C | C | A | A | B | A | A | **Reasons of abandoned surgery** |
| 31/  AWD | 25/  FOD^&^ | 24/  AWD | 24/  AWD | 21/DOD | 21/DOD | 43/DOD | **OS (ms)/ status** |

**Supplementary Table 2.** Characteristics of studies included in the discussion.

**Abbreviations:** LAACC, locally advanced adherent colon cancer; LRACC, locally recurrent adherent colon cancer; LASCC, locally advanced sigmoid colon cancer; LACC, locally advanced colon cancer; PC, postsurgical complications; NRT, neoadjuvant Radiotherapy; NCRT, neoadjuvant chemoradiotherapy; GTV, gross tumor volume; XELIRI, irinotecan and capecitabine.

| The current study | XXX,  et al.(XXX)^(13)^ | XXX, et al.(XXX)^(12)^ | Hawkins,  et al.(2019)^(23)^ | | Krishnamurty,  et al. (2018)^(22)^ | | Huang,  et al.(2017)^(21)^ | | Hallet,  et al.(2014)^(20)^ | | Cukier et al,(2012)^(22)^ | | **Study** |
| --- | --- | --- | --- | --- | --- | --- | --- | --- | --- | --- | --- | --- | --- |
| China | China | China | USA | | USA | | Taiwan | | Canada | | Canada | | **Country** |
| Unresectable LACC | Unresectable LACC | unresectable LASCC | LACC  (cT4) | | LACC  (cT4） | | LACC | | LRACC | | LAACC | | **Disease** |
| 106 | 60 | 21 | 15012 | 195 | 108 | 23 | 36 | | 15 | | 33 | | **Number of patients** |
| NCRT | NCRT | NRT | non-NRT | NRT | non-NRT | NRT | NCRT | | NCRT+  MVR | | NCRT+  MVR | | **Treatment strategy** |
| GTV (45-54Gy/23-27F) | GTV (46-50 GY/23-27F) | GTV (46-50 GY/25-28F) | GTV (45GY/25F） | | GTV (39-50.4 GY/25F) | | GTV (45-50.4 GY/25-26F) | | GTV (45-50 GY/25F) | | GTV (45-50 GY/25F) | | **RT scheme** |
| mFOLFOX6  CAPEOX /XELIRI | mFOLFOX6  CAPEOX /XELIRI | Capecitabine-based regimen |  | | FOLFOX | | FOLFOX | | 5Fu  225 mg/m^2^/day | | 5FU 225  mg/m 2 /day | | **CT scheme** |
| 88.5 | 93 |  |  | | 6.50 | 30.4 | 85.3 | |  | |  | | **Downstage rate** |
| 88.8 | 86 | 95.2 | 79.8 | 87.2 | 88 | 95.7 |  | | 86.7 | | 100% | | **R0 rate** |
| 22.4 | 26.3 | 38.1 |  | |  | | 26.4 | | 6.7 | | 3 | | **pCR rate** |
| 6.1% | 7% | 0% |  |  | 8.3% | 17.4% | 14.7% | | 20% | | 36% | | **PC**  **(Grade 3-4)** |
| 35.5m | 26m | 42m | 36.1m | | 52.6m | | 23.5m | | 54m | | 36m | | **Follow-up** |
| 3y OS | 26m OS | 3y OS | 5y OS | | 5y OS | | 2y DFS | 2y OS | 5y DFS | 5y OS | 3Y DFS | 3y OS | **Results** |
| 82.1% | 76.7% | 95.2% | 45.7% | 62% | 51.5% | 88.7% | 63.5% | 90% | 73.7% | 85.9% | 73.1% | 85.9% |  |

**Supplementary Table 3.** Univariate Cox analysis of prognostic factors for OS, PFS, DFS.

| **Variables** | **P value** | | |
| --- | --- | --- | --- |
|  | **OS** | **PFS** | **DFS** |
| Gender (female vs male) | 0.839 | 0.639 | 0.337 |
| age(≤65y vs >65y） | 0.549 | 0.901 | 0.716 |
| BMI | 0.947 | 0.936 | 0.812 |
| BMI<18.5 | - | - | - |
| 18.5≤BMI<24 | 0.838 | 0.816 | 0.573 |
| BMI≥24 | 0.742 | 0.717 | 0.526 |
| KPS (80 vs 90) | 0.349 | 0.033 | 0.027 |
| Site (sigmoid vs non-sigmoid) | 0.586 | 0.859 | 0.886 |
| Differentiation | 0.025 | 0.194 | 0.117 |
| high | - | - | - |
| middle | 0.694 | 0.804 | 0.233 |
| low | 0.042 | 0.125 | 0.048 |
| CEA level(≤5ng/ml vs >5ng/ml） | 0.425 | 0.349 | 0.325 |
| Complication (no vs yes) | 0.192 | 0.198 | 0.221 |
| cT stage (cT3-4a vs cT4b) | 0.919 | 0.463 | 0.376 |
| cN stage (N0-1 vs N2) | 0.232 | 0.921 | 0.603 |
| cTNM stage (IIC + IIIB vs IIIC) | 0.646 | 0.237 | 0.338 |
| ypT stage | <0.001 | <0.001 | 0.121 |
| ypT0-T3 | - | - |  |
| ypT4a-4b | 0.031 | 0.138 |  |
| NRS | <0.001 | <0.001 | - |
| ypN stage | <0.001 | <0.001 | 0.381 |
| pN0 | - | - |  |
| pN1-2 | 0.596 | 0.455 |  |
| NRS | <0.001 | <0.001 | - |
| ypTNM stage | <0.001 | <0.001 | 0.071 |
| yp0-IIA | - | - |  |
| ypIIB-IIIC | 0.043 | 0.070 |  |
| NRS | <0.001 | <0.001 | - |
| pCR (pCR vs non-pCR) | <0.001 | 0.001 | 0.156 |
| pCR | - | - |  |
| Non-pCR | 0.250 | 0.127 |  |
| NRS | <0.001 | 0.001 |  |
| MVR | <0.001 | <0.001 | 0.775 |
| Non-MVR | - | - |  |
| MVR | 0.616 | 0.810 |  |
| NRS | <0.001 | <0.001 | - |
| Resection group (non-R0 vs R0) | <0.001 | <0.001 | - |
| MMR (dMMR vs pMMR) | 0.880 | 0.754 | 0.832 |
| VLPNI (negative vs positive) | 0.002 | 0.004 | 0.004 |
| Resection Surgery-Radiotherapy interval (≤63d vs >63d) | 0.266 | 0.458 | 0.258 |

**Supplemental Table 4.1** Multiple linear regression coefficients

| **Variables** | **VIF** | **tolerance** |
| --- | --- | --- |
| Gender | 1.3 | 0.787 |
| Age | 1.4 | 0.736 |
| BMI | 1.7 | 0.585 |
| KPS | 1.4 | 0.722 |
| Site | 2.2 | 0.460 |
| CEA-pre level | 1.5 | 0.690 |
| Differentiation | 1.8 | 0.566 |
| Complication | 1.5 | 0.671 |
| cT stage | 2.3 | 0.431 |
| cN stage | 1.8 | 0.558 |
| cTNM stage | 1.7 | 0.585 |
| ypT stage | 19.4 | 0.051 |
| ypN stage | 3.4 | 0.294 |
| ypTNM stage | 24.0 | 0.042 |
| pCR | 1.6 | 0.627 |
| MMR | 1.9 | 0.525 |
| MVR | 1.7 | 0.588 |
| VLPNI | 1.9 | 0.531 |
| R0 resection | 1.4 | 0.703 |
| Resection Surgery-Radiotherapy interval | 1.8 | 0.557 |

Abbreviations: VIF:variance inflation factor;

**Supplemental Table 4.2** Correlation matrix analysis:

| **cTNM stage** | **cN stage** | **cT stage** | **Complication** | **CEA** | **Differentiation** | **Site** | **KPS** | **BMI** | **Age** | **Gender** | **Correlation coefficient** |
| --- | --- | --- | --- | --- | --- | --- | --- | --- | --- | --- | --- |
|  |  |  |  |  |  |  |  |  |  | 1 | **Gender** |
|  |  |  |  |  |  |  |  |  | 1 | 0.048 | **Age** |
|  |  |  |  |  |  |  |  | 1 | 0.056 | 0.181 | **BMI** |
|  |  |  |  |  |  |  | 1 | -0.218 | -0.187 | 0.075 | **KPS** |
|  |  |  |  |  |  | 1 | 0.057 | -0.160 | 0.153 | 0.098 | **Site** |
|  |  |  |  |  | 1 | -0.281 | -0.178 | 0.032 | 0.090 | -0.057 | **Differentiation** |
|  |  |  |  | 1 | -0.017 | 0.077 | 0.023 | 0.216 | 0.101 | 0.051 | **CEA** |
|  |  |  | 1 | -0.016 | 0.017 | -0.149 | -0.023 | 0.266 | -0.015 | -0.051 | **Complication** |
|  |  | 1 | -0.156 | -0.121 | 0.057 | 0.283 | -0.104 | -0.234 | 0.233 | -0.093 | **cT stage** |
|  | 1 | 0.131 | 0.149 | 0.069 | 0.224 | 0.049 | 0.002 | 0.011 | 0.031 | -0.091 | **cN stage** |
| 1 | 0.416 | 0.396 | 0.029 | -0.029 | 0.305 | 0.097 | -0.104 | 0.003 | 0.120 | -0.194 | **cTNM stage** |
| 0.277 | 0.097 | 0.277 | 0.006 | -0.291 | 0.154 | 0.153 | -0.147 | -0.206 | 0.108 | -0.175 | **ypT stage** |
| -0.022 | -0.024 | -0.022 | -0.019 | 0.019 | 0.073 | -0.128 | -0.165 | 0.264 | -0.157 | 0.063 | **ypN stage** |
| 0.213 | 0.149 | 0.213 | 0.016 | -0.227 | 0.080 | 0.207 | -0.112 | -0.156 | 0.071 | -0.128 | **ypTNM stage** |
| 0.100 | -0.01 | -0.107 | 0.203 | -0.203 | 0.17 | 0.129 | -0.113 | -0.093 | -0.073 | -0.150 | **pCR** |
| 0.198 | -0.154 | 0.381 | 0.005 | -0.005 | -0.067 | 0.275 | -0.027 | 0.054 | 0.068 | -0.016 | **MVR** |
| -0.124 | -0.205 | -0.124 | -0.175 | 0.175 | 0.034 | 0.058 | -0.132 | 0.049 | 0.139 | -0.026 | **R0 Resection** |
| -0.136 | -0.061 | -0.026 | 0.134 | 0.117 | -0.083 | 0.156 | 0.267 | -0.190 | 0.275 | -0.074 | **MMR** |
| 0.120 | 0.031 | -0.218 | 0.071 | 0.015 | 0.321 | 0.066 | -0.079 | 0.056 | -0.051 | -0.045 | **VLPNI** |
| 0.048 | 0.042 | -0.044 | 0.155 | 0.126 | 0.175 | -0.164 | -0.171 | -0.139 | 0.033 | -0.047 | **Resection Surgery-Radiotherapy interval** |

**Supplemental table 4.2**(continue):

This table would be added to the section “**Supplement Materials**” section.

| **Resection Surgery-Radiotherapy**  **interval** | **VLPNI** | **MMR** | **R0 Resection** | **MVR** | **pCR** | **ypTNM stage** | **ypN stage** | **ypT stage** | **Correlation coefficient** |
| --- | --- | --- | --- | --- | --- | --- | --- | --- | --- |
|  |  |  |  |  |  |  |  | 1 | **ypT stage** |
|  |  |  |  |  |  |  | 1 | 0.128 | **ypN stage** |
|  |  |  |  |  |  | 1 | 0.353 | 0.931 | **ypTNM stage** |
|  |  |  |  |  | 1 | 0.203 | 0.072 | 0.189 | **pCR** |
|  |  |  |  | 1 | 0.086 | 0.284 | 0.051 | 0.289 | **MVR** |
|  |  |  | 1 | 0.009 | -0.064 | -0.175 | 0.084 | -0.197 | **R0 Resection** |
|  |  | 1 | 0.017 | -0.106 | -0.126 | -0.033 | -0.424 | 0.013 | **MMR** |
|  | 1 | -0.236 | 0.139 | -0.018 | 0.119 | 0.329 | 0.298 | 0.282 | **VLPNI** |
| 1 | 0.204 | 0.014 | 0.165 | -0.108 | 0.259 | 0.295 | 0.153 | 0.235 | **Resection Surgery-Radiotherapy interval** |
